# *Agrobacterium*-Mediated Gene Transient Overexpression and *Tobacco Rattle Virus* (TRV)-Based Gene Silencing in Cassava

**DOI:** 10.3390/ijms20163976

**Published:** 2019-08-15

**Authors:** Hongqiu Zeng, Yanwei Xie, Guoyin Liu, Yunxie Wei, Wei Hu, Haitao Shi

**Affiliations:** 1 Hainan Key Laboratory for Sustainable Utilization of Tropical Bioresources, College of Tropical Crops, Hainan University, Haikou 570228, China; 2 Key Laboratory of Biology and Genetic Resources of Tropical Crops, Institute of Tropical Bioscience and Biotechnology, Chinese Academy of Tropical Agricultural Sciences, Xueyuan Road 4, Haikou 571101, China

**Keywords:** *agrobacterium*, transient expression, virus-induced gene silencing (VIGS), *tobacco rattle virus* (TRV), cassava (*Manihot esculenta*)

## Abstract

*Agrobacterium*-mediated transient expression and virus-induced gene silencing (VIGS) are very useful in functional genomics in plants. However, whether these methods are effective in cassava (*Manihot esculenta*), one of the most important tropical crops, remains elusive. In this study, we used *green fluorescent protein* (*GFP*) and *β-glucuronidase* (*GUS*) as reporter genes in a transient expression assay. *GFP* or *GUS* could be detected in the infiltrated leaves at 2 days postinfiltration (dpi) and were evidenced by visual *GFP* and *GUS* assays, reverse-transcription PCR, and Western blot. In addition, *phytoene desaturase* (*PDS*) was used to show the silencing effect in a VIGS system. Both *Agrobacterium* GV3101 and AGL-1 with *tobacco rattle virus* (TRV)-*MePDS*-infiltrated distal leaves showed an albino phenotype at 20 dpi; in particular, the AGL-1-infiltrated plants showed an obvious albino area in the most distal leaves. Moreover, the silencing effect was validated by molecular identification. Notably, compared with the obvious cassava mosaic disease symptom infiltrated by *African*-*cassava*-*mosaic*-*virus*-based VIGS systems in previous studies, TRV-based VIGS-system-infiltrated cassava plants did not show obvious virus-induced disease symptoms, suggesting a significant advantage. Taken together, these methods could promote functional genomics in cassava.

## 1. Introduction

Aiming at analyzing genomic sequences and functions, functional genomics has rapidly developed as a result of sequencing projects of different species, especially of important crops. Cassava (*Manihot esculenta*), a kind of tuber crop, is widely cultivated in the tropics and some subtropical areas. Because of its low planting cost and high efficiency, it has become one of the most important industrial crops in the world [1]. Although cassava sequencing and resequencing were accomplished years ago, cassava functional genomics has developed slowly due to the time-consuming nature and low transformation rate of obtaining stable transgenic cassava plants [2]. Therefore, it is necessary to find another way to promote rapid gene function studies of cassava.

*Agrobacterium*-mediated transient overexpression is widely used in gene function studies in plants [3]. The most important advantages of this method are its rapid and simple process and that it does not require complex equipment, unlike other overexpression assays [4]. The simple protocol is accomplished by creating an overexpression vector, transforming it into *Agrobacterium*, and infiltrating the *Agrobacterium* into plant culture [5]. *Green fluorescent protein* (*GFP*) and *β-glucuronidase* (*GUS*) are two convenient reporter genes in biology which can be observed by fluorescent microscopy or direct staining [6,7]. Normally, they can continue to express, reaching their peak at 3 days postinfiltration (dpi), and exert obvious expression for at least one week [4,8]. In addition, they can be efficiently expressed in a wide range of plant species. So far, transient overexpression assays have been successfully used in many species, such as *Arabidopsis* spp. [4], tobacco [5], *Mimulus lewisii* [6], *Piper colubrinum* [7], rose [9], soybean [10], *Theobroma cacao* L. [11], cotton [12], *Brassica juncea* [13], potato [14], tomato [15], and lettuce [15]. However, its effectiveness in cassava requires further investigation.

*Agrobacterium*-mediated virus-induced gene silencing (VIGS) is a widely used, efficient technique in gene function studies [16]. VIGS is mostly based on RNA viruses, such as *tobacco mosaic virus* (TMV), *potato virus X* (PVX), *barley stripe mosaic virus* (BSMV), *cucumber mosaic virus* (CMV), and *tobacco rattle virus* (TRV) [17,18,19,20,21,22,23]. Among these viruses, TRV is widely used because of its high silencing effect, long silencing duration, wide range of infiltration host species, and the mild virus-induced disease symptoms [24,25]. Presently, TRV has been used in *Arabidopsis* [26], tobacco [27], strawberry [27,28], cotton [29], piper [30], wheat [31], maize [31], and so on. For the silencing effect, most of the abovementioned infiltrated species show the phenotype at two weeks postinfiltration and the most obvious changes around the third week [27,29,32].

In this study, *Agrobacterium*-mediated transient overexpression and VIGS were established in cassava. The phenotype and examination of these assays illustrated the feasibility of the protocols, which should help promote the rapid analysis of functional genomics in cassava.

## 2. Results

### 2.1. The Effect of the Transient Expression Assay in Cassava Leaves

**Figure 1:**
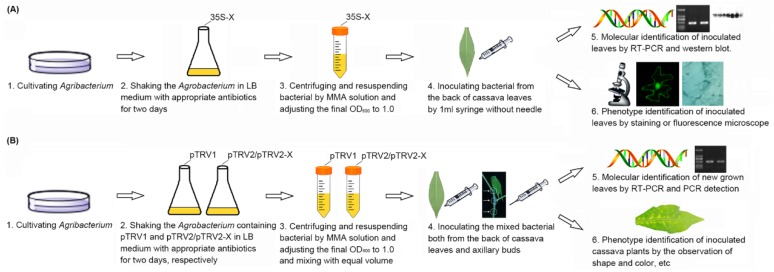
Schematic views of *Agrobacterium*-mediated gene transient expression and TRV based gene silencing systems in cassava. (**A**) The schematic view of *Agrobacterium*-mediated gene transient expression assay. (**B**) The schematic view of *Agrobacterium*-mediated TRV based VIGS assay. The white arrows and circles pointed the axillary buds. The figure was drew by using ChemDraw (https://chemdrawdirect.perkinelmer.cloud/js/sample/index.html) and Microsoft Office PowerPoint 2003 (Microsoft, Redmond, WA, USA).

**Figure 2:**
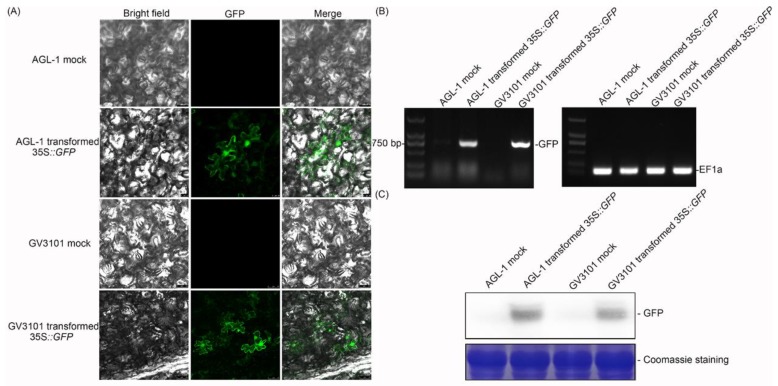
Analysis of transient expression of *green fluorescent protein* (*GFP*) in cassava leaves. (**A**) At 2 days postinfiltration (dpi), *Agrobacterium* with no plasmid was used as the mock, and *Agrobacterium* containing *35S::GFP* was used as the sample. Bar = 25 μm. (**B**) The relative transcript level of *GFP* is shown by reverse-transcription PCR, and *elongation factor 1* (*EF1*) was used as a reference gene. (**C**) *GFP* was detected by Western blot assay, 10 μg total protein extracted from infiltrated leaves were loaded onto SDS-PAGE gel, and Coomassie staining shows the equal protein content.

To investigate the effect of *GFP* transient expression, the *35S::GFP* vector [33] was transformed into *Agrobacterium* AGL-1 and GV3101, and the transformed strains were infiltrated into cassava leaves, respectively (Figure 1). At 2 dpi, the infiltrated leaves were harvested and examined by confocal laser-scanning microscopy. *Agrobacterium* with *35S::GFP*-infiltrated leaves showed clear green fluorescence in both the cytoplasm and nucleus, while the empty *Agrobacterium*-infiltrated leaves showed no fluorescence (Figure 2A). Reverse-transcription PCR was performed to clarify the effect of transient expression. *Agrobacterium* with the *35S::GFP*-infiltrated sample showed a bright band by PCR, indicating that *GFP* was expressed in the cassava leaves (Figure 2B). Moreover, *GFP* was also detected by Western blot, which was consistent with confocal observation (Figure 2C). 

**Figure 3:**
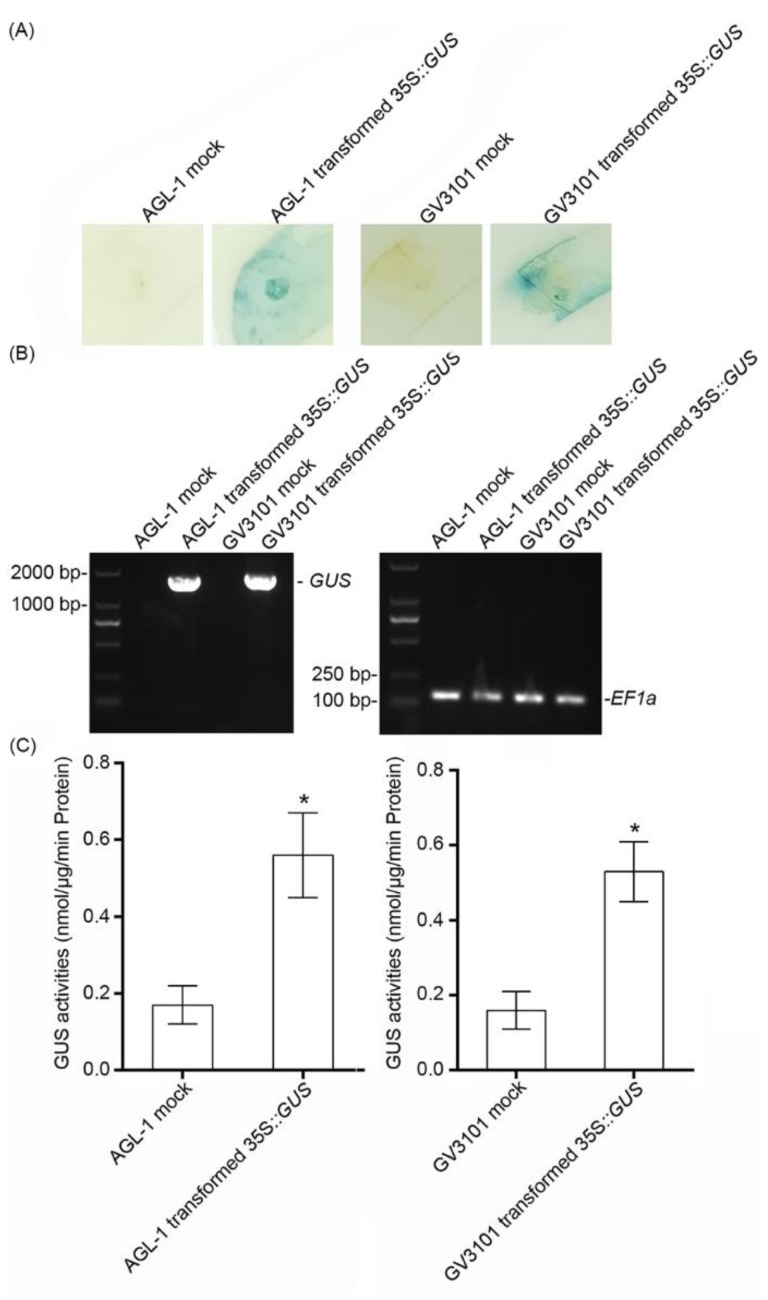
Analysis of transient expression of *β-glucuronidase* (*GUS*) in cassava leaves. (**A**) At 2 dpi, *Agrobacterium* with no plasmid was used as the mock, and *Agrobacterium* containing *35S::GUS* was used as the sample. The leaves were stained using *GUS* staining solution. Bar = 1 cm. (**B**) The relative transcript level of *GUS* is shown by reverse-transcription PCR, and *EF1* was used as a reference gene. (**C**) The *GUS* activities of infiltrated leaves. Statistical tests were performed using IBM SPSS (v21). Briefly, the Kolmogorov–Smirnov test and Levene’s test were performed to check the normality of the data distribution and the homogeneity of variance of the data, respectively. The statistical analysis was performed using Student’s *t*-test and the Tukey–Kramer test. Asterisk symbol (*) indicates significant difference at *p* < 0.05. *n* = 10 per group. All data are expressed as mean ± SD of three independent experiments.

To further confirm the effect of transient expression, *GUS* was used as the second marker. After *GUS* staining, *Agrobacterium* with the *35S::GUS*-infiltrated area became light blue (Figure 3A), indicating that *GUS* was expressed at the protein level. In addition, reverse-transcription PCR showed that the expression of *35S::GUS* could be examined at the transcript level (Figure 3B), with significant *GUS* activity (Figure 3C). These results suggested that *GFP* and *GUS* were expressed with biological activity in the transient expression assay. 

Based on the above results of *GFP* and *GUS* overexpression, the effects of transient overexpression showed no significant difference in *GFP* expression and *GUS* activity between AGL-1 and GV3101 (Figure 2 and Figure 3, Table 1).

### 2.2. Silencing of MePDS in Cassava

**Figure 4:**
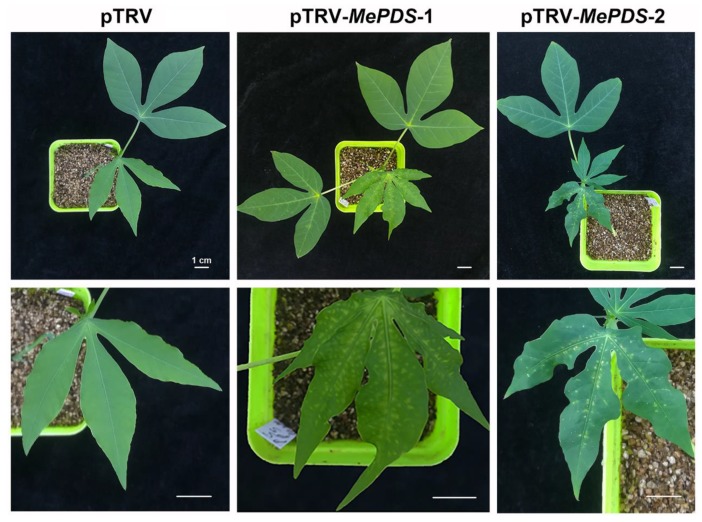
Phenotypes of the infiltrated albino cassava plants. The cassava plants were infiltrated with *Agrobacterium* strain AGL-1 containing the pTRV1 and pTRV2 vectors, which were used as the mock, pTRV1 and pTRV2-*MePDS*, which were used to test the albino effect. In the photos, the upper line shows the whole cassava plants and the underline shows the magnified leaves, both at 20 dpi. Bar = 1 cm.

To reveal the effect of TRV-based VIGS on cassava, *phytoene desaturase* (*PDS*) was selected as a reporter gene. A 370 bp sequence of *MePDS* was amplified and inserted into the multiple cloning site of the pTRV2 vector. The pTRV1, pTRV2, and pTRV2-*MePDS* vectors were transformed into *Agrobacterium*, respectively. The cultivated *Agrobacterium* solution was infiltrated into cassava local leaves and axillary buds (Figure 1). At 20 dpi, AGL-1-infiltrated cassava showed an obvious albino area in the distal leaves, especially for the area around the main vein (Figure 4).

**Figure 5:**
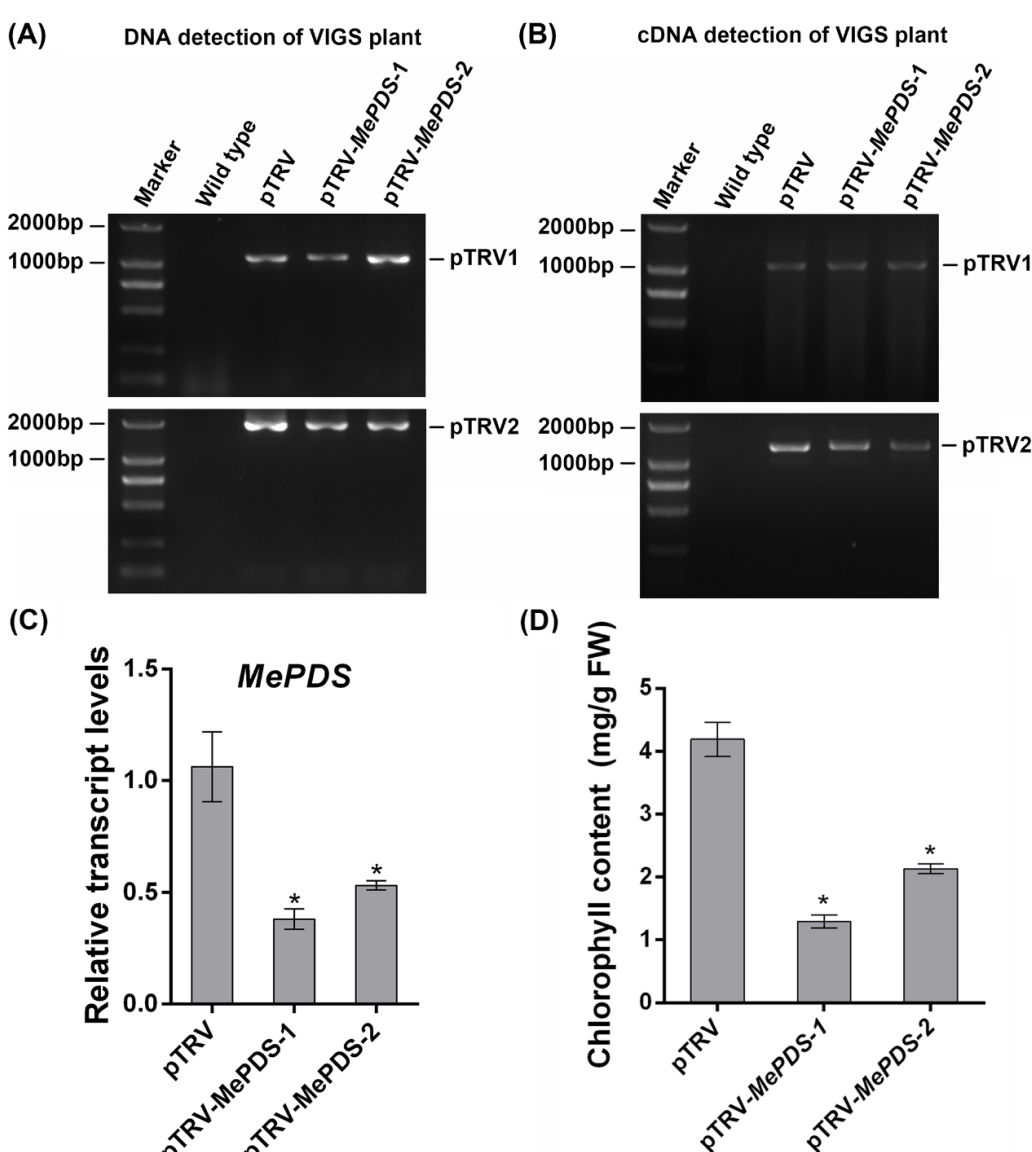
Detection of VIGS cassava plants. (**A**) PCR detection of pTRV1 and pTRV2 sequences from the genomic DNA of VIGS cassava plants. (**B**) Reverse-transcription PCR detection of pTRV1 and pTRV2 sequences from the cDNA of VIGS cassava plants. (**C**) The relative transcript levels of *MePDS* of the infiltrated cassava plants. *MeEF1* was used as internal control. (**D**) The chlorophyll contents in the infiltrated cassava plants. Statistical tests were performed using IBM SPSS (v21). Briefly, the Kolmogorov–Smirnov test and Levene’s test were performed to check the normality of the data distribution and the homogeneity of variance of the data, respectively. The statistical analysis was performed using Student’s *t*-test and the Tukey–Kramer test. Asterisk symbol (*) indicates significant difference at *p* < 0.05. All data are expressed as mean ± SD of three independent experiments.

To further investigate the level of *MePDS* silencing, DNA was extracted from distal leaves and sequences of pTRV1, pTRV2, or pTRV2-*MePDS* were detected by PCR. The result indicated that the sequences of pTRV vectors could be detected in the distal leaves of all AGL-1-infitrated cassava plants but not in the wild-type (WT) cassava (Figure 5A). In addition, the transcript levels of pTRV1 and pTRV2 sequences in the distal leaves were analyzed. Both the transcripts of pTRV1 and pTRV2 could be obviously examined in the pTRV and pTRV-*MePDS* cassava plants but showed no PCR band in WT cassava (Figure 5B). Notably, the pTRV-*MePDS* cassava leaves showed an obvious albino area around the main vein. The transcript level of *MePDS* in the distal leaves of *MePDS*-VIGS plants was 37.9% and 53.1% of that in the mock (Figure 5C), displaying significantly lower chlorophyll content than the mock (Figure 5D). Based on these results, we could conclude that the gene silencing of *MePDS* in the distal leaves of *MePDS*-VIGS plants was caused by VIGS, and the albino phenotype in the distal leaves was derived by VIGS but not the infection-induced effects, which resulted in no significant difference in local leaves.

Compared with the phenotype of *Agrobacterium* AGL-1-infiltrated distal leaves, GV3101-infiltrated distal leaves showed the albino phenotype in the part around the petiole (Supplementary Figure S1), with slightly weaker PCR detection bands, a lower *MePDS* transcript level, and lower chlorophyll content than the mock (Supplementary Figure S2). For AGL-1, 75.00% of cassava plants showed less than 60% transcript level of *MePDS* in the distal leaves, and only 37.50% of the plants exhibited the albino phenotype (Table 2). For GV3101, 62.50% of cassava plants showed a lower transcript level of *MePDS* in the distal leaves, and only 12.50% of plants showed the albino phenotype (Table 2). These results suggested that both AGL-1 and GV3101 with TRV could induce *MePDS* silencing and regulate the color of leaves in cassava, with better effects in AGL-1-transformed TRV-based VIGS. 

## 3. Discussion

Although genome sequencing of cassava has been completed for several years now, little progress has been made in determining the functional genomics of cassava [34]. In order to address this question and promote functional genomics in cassava, this study investigated *Agrobacterium*-mediated gene transient overexpression and TRV-based VIGS. 

The *Agrobacterium*-mediated transient overexpression assay is a powerful tool for analyzing gene function in vivo [35]. In this study, *Agrobacterium* AGL-1 and GV3101 with *GFP* or *GUS* overexpressing plasmids were used for transient overexpression. In a previous study, GV3101 containing the *35S::GUS* vector was used in MCOL2215 and 60,444 cassava varieties to test the transient expression effect, but no *GUS* was detected. However, AGL with the *35S::GUS* vector showed a positive result [36]. In this study, both *GFP* and *GUS* were expressed by the GV3101 strain in SC124 and could be detected at the transcript (Figure 2 and Figure 3) and protein (Figure 2 and Figure 3) levels, indicating that they could be efficiently expressed in cassava leaves. Generally, the GV3101- and AGL-1-based transient overexpression assays had relatively high success rates (Table 1), while the negative results might have been due to the RNA cosilence process [37,38], the morphology, or the structure of the plants [39]. Therefore, whether the target genes are overexpressed in the transient overexpression assay should be analyzed first. 

In addition, *Agrobacterium*-mediated VIGS is another powerful tool to investigate gene function in vivo. Different *Agrobacterium* strains (GV3101 and AGL-1) with pTRV vectors were used to silence the *MePDS* gene. AGL-1 with the transformation of pTRV1 and pTRV2-*MePDS* vector-infiltrated distal leaves showed obvious albino phenotypes at 20 dpi (Figure 4). Interestingly, the albino phenotype only showed in distal leaves and the albino area was mainly located around the leaf vein, similar to the results in *California poppy* [40] and *Solanum pseudocapsicum* [41]. From the albino leaf blade, we found that the area near the petiole became white, while the area distant from the petiole was still green (Figure 4), indicating that TRV-based *MePDS* silence might spread from the bottom to the tip of the blade in cassava. Additionally, TRV-based *Agrobacterium*-mediated VIGS was examined by genome DNA or cDNA to ensure the *Agrobacterium*-mediated transformation and expression of pTRV1 and pTRV2 or pTRV2-X [41,42]. PCR results indicated that *Agrobacterium*-mediated pTRV1 and pTRV2 or pTRV2-*MePDS* could be successfully transformed and expressed in the distal leaves of cassava plants, with a 37.9%–53.1% transcript level of *MePDS* and 58%–76.5% of chlorophyll content in the distal plant leaves (Figure 5). Because the relative transcript level of *MePDS* of the albino cassava plants was 71% of the mock and the detected bands were weak, GV3101 with pTRV1 and pTRV2-*MePDS* infiltrated cassava plants showed less obvious change, and only the part around the petiole became albino (Supplementary Figures S1 and S2). VIGS is a post-transcriptional gene silencing method [24,25]. In a VIGS system, the expressed levels of silenced marker genes were 30%–40% of control in *Solanum pseudocapsicum* L. [41], 37% of control in columbine [43], 41%–60% of control in barley [44], 28%–38% of control in tomato [45], and 30%–70% of control in rose [27]. Although these genes could not silenced completely, the albino phenotype was obvious. Consistently, the transcript level of *MePDS* in *MePDS*-VIGS plants were 37.9%–53.1% of that in the mock, with an obvious albino phenotype and a 58%–76.5% level of chlorophyll. Compared with GV3101, *Agrobacterium* AGL-1 showed better effects in the VIGS system (Table 2), which might have been due to the specific hypervirulence of AGL-1 [46]. AGL-1 is derived from pTiBo542, which has a high induction of the *vir* gene [47], which is necessary for T-DNA transfer [48].

In this study, an *Agrobacterium*-mediated transient overexpression system was established in cassava. The successful examination of the transcription, translation, and biological activity of expressed proteins suggests its application in subcellular localization experiments, bimolecular fluorescence complementation (BIFC), co-immunoprecipitation (Co-IP), enzyme activity detection, and so on. In addition, a TRV-based *Agrobacterium*-mediated VIGS system was also successfully verified in cassava (Figure 1B), as shown by the expressions of pTRV and the corresponding gene, as well as the albino phenotype. Previous studies have found that cassava could be silenced by an *African*-*cassava*-*mosaic*-*virus*-based VIGS system; however, this could lead to serious malformation of newly grown leaves, thereby resulting in interference in the assay of plant disease resistance [49,50,51]. On the contrary, TRV-based VIGS-system-infiltrated cassava plants only showed mild virus-induced disease symptoms and might be more appropriate for gene function analysis. In conclusion, this study provided two valid and efficient methods for the characterization of gene function, so as to promote functional genomics in cassava.

## 4. Materials and Methods

### 4.1. Plant Materials

The cassava plants South China 124 (SC124) were kindly provided by Dr. Wei Hu (Institute of Tropical Bioscience and Biotechnology, Haikou, China). Two-week-old tissue culture cassava plants (SC124) were transferred to small pots and grown in a chamber for two weeks until the experiment at 25 ℃ under a 16 h light/8 h dark cycle. 

### 4.2. Vectors and Vector Construction

The pEGAD and pBI121 vectors were used to express *GFP* and *GUS*, respectively. The full-length sequences of *GFP* and *GUS* were driven by the 35S promoter. The VIGS assay used pTRV1 and pTRV2 vectors, which have been described previously [52]. The partial sequence of the *MePDS* gene was cloned into the multiple cloning site of the pTRV2 vector, and the primers are listed in Supplementary Table S1.

### 4.3. Agrobacterium Infiltration of Cassava

*Agrobacterium* strains GV3101 and AGL-1 were transformed by different plasmids: pEGAD, pBI121, pTRV1, pTRV2, and pTRV2-*MePDS*, respectively. GV3101 was cultivated in liquid LB medium containing 50 mg/L kanamycin, 20 mg/L rifampicin, and 50 mg/L gentamycin, while AGL-1 was cultivated in LB medium with 50 mg/L kanamycin, 20 mg/L rifampicin, and 50 mg/L carbenicillin. Both were then shook at 28 ℃ at 200 rpm for 2 days. The bacteria was centrifuged at 4000 rpm for 10 min, then the supernatant was discarded. The remaining bacteria was washed with double-distilled water, then centrifuged, and the supernatant was discarded again. The bacterial sediment was resuspended in MMA solution (10 mM MgCl, 10 mM MES, and 150 μM acetosyringone), the OD_600_ was adjusted to 1, and then the resuspended bacterial solution was placed in the dark for 3 h. For the transient expression assay, the standing bacterial solution was infiltrated into the second and third leaves from the top of the cassava by a 1 mL needle. For the VIGS assay, the bacterial solutions containing pTRV1 or pTRV2-*MePDS* were mixed with the same volume, and the mixture of pTRV1 or pTRV2 was used as the mock. The mixed solution was infiltrated into both the leaves and axillary buds of cassava plants to keep the silencing effect [48]. After infiltration, the cassava plants were removed to the chamber at the same light and temperature.

### 4.4. DNA and RNA Extraction 

DNA was extracted by the Plant Genomic DNA Extraction Kit (DP305, TIANGEN, Beijing, China). Total RNA was extracted by the RNAprep Pure Plant Kit (Polysaccharides & Polyphenolics-rich) (DP441, TIANGEN, Beijing, China). The remaining DNA from the extracted RNA was digested by RNase-free DNase I (EN0521, Thermo, Waltham, MA, USA). Then, the quality and concentration of DNA and RNA were examined by Nano Drop 2000 (Thermo, Waltham, MA, USA).

### 4.5. Reverse-Transcription PCR and Quantitative Real-Time PCR

The first strand cDNA was synthesized by the RevertAid First Strand cDNA Synthesis Kit (K1621, Thermo, Waltham, MA, USA). Then, the cDNA was adjusted to an equal concentration. Reverse-transcription PCR was performed by EasyTaq PCR SuperMix (AS111, TRANS, Beijing, China) with a PCR program of (1) 94 ℃ for 3 min; (2) 29 cycles of 94 ℃ for 30 s, 55 ℃ for 30 s, and 72 ℃ for 2 min; and (3) 72 ℃ for 10 min and 16 ℃ for storage.

Quantitative real-time PCR was performed using TransStart Tip Green qPCR SuperMix (AQ141, TRANS, Beijing, China) in LightCycler® 96 (Roche, Basel, Switzerland) with a PCR program of (1) 94 ℃ for 30 s; (2) 45 cycles of 94 ℃ for 5 s, 55 ℃ for 15 s, and 72 ℃ for 15 s; and (3) 95 ℃ for 10 s, 65 ℃ for 60 s, and 97 ℃ for 1 s as the melting curve. *Elongation factor 1* (*EF1*) was used as the reference gene, and the primers used in reverse-transcription PCR and quantitative real-time PCR are listed in Supplementary Tables S2 and S3.

### 4.6. Confocal Microscopy Scanning

At 2 dpi, the infiltrated area of cassava leaves was harvested, cut into small pieces, and then observed by confocal laser-scanning microscopy (TCS SP8, Leica, Heidelberg, Germany).

### 4.7. Protein Extraction and Western Blot

Cassava leaves were harvested and ground by liquid nitrogen and phosphate buffer solution (pH 7.4). The extracted solution was centrifuged at 12,000 rpm and 4 ℃ for 10 min. The supernatant was boiled with SDS-PAGE sample loading buffer (P0015, Biyotime, Shanghai, China) for 5 min. After centrifugation for 10 min, the supernatant could be used in Western blot. The Western blot assay was performed according to a previous study [53]. Briefly, the protein samples were loaded into 12% polyacrylamide gel and separated by electrophoresis. The polyacrylamide gel was transferred to a PVDF membrane (475855-1R, Millpore, Massachusetts, America) by a Trans-Blot SD Semi-Dry Electrophoretic Transfer Cell (1703940, Bio-rad, Hercules, America). Then, the PVDF membrane was blocked and incubated in 5% skim milk with anti-*GFP* antibody (AG281, Biyotime, Haimen, China).

### 4.8. GUS Staining and Activity Detection

*GUS* staining and *GUS* activity assay were carried out according to a previous study with slight modifications [48]. The infiltrated cassava leaves were harvested and immersed into *GUS* staining solution (50 mM NaH_2_PO_4_, 50 mM Na_2_HPO_4_, 10 mM EDTA-Na_2_, 0.5 mM K_4_[Fe(CN)_6_], 0.5 mM K_3_[Fe(CN)_6_], 0.1% Triton-X100, and 2 mM X-Gluc; pH 7.0) with vacuum infiltration for 0.5 h in the dark, and then the plant leaves were incubated in the dark at 37 ℃ for at least 12 h. After staining, the leaves were immersed into 70% alcohol to remove chlorophyll.

The infiltrated leaves were harvested and ground by liquid nitrogen and phosphate buffer solution (pH 7.4). The extracted solution was centrifuged (12,000 rpm, 10 min, 4 ℃) and the supernatant was used for further assay. The extraction was incubated in 1 mM 4-Methylumbelliferyl-b-D-glucuronide (4-MUG) at 37 ℃, the reaction mixture was taken out per 5 min, and Na_2_CO_3_ was added to stop the reaction. The samples were detected by a microplate system (Infinite M200 Pro, TECAN, Hombrechtikon, Switzerland) with 365 nm excitation and 455 nm emission. The *GUS* activity was calculated by the standard curve made by different concentrations of 4-methylumbelliferone (4-MU).

### 4.9. PCR Detection of Cassava Plants 

The transient expressed cassava plants were detected by PCR using genomic DNA and cDNA, with a PCR program of (1) 94 ℃ for 3 min; (2) 29 cycles of 94 ℃ for 30 s, 55 ℃ for 30 s, and 72 ℃ for 2 min; and (3) 72 ℃ for 10 min and 16 ℃ for storage. The primers are listed in Supplementary Table S4.

### 4.10. Chlorophyll Content of Cassava Leaves

The chlorophyll content of the cassava leaves was quantified as previously described [54]. The harvested leaves were ground in 80% acetone. After centrifugation, the absorbance of OD_647_ and OD_665_ of the supernatant solution was detected by a microplate system (Infinite M200 Pro, TECAN, Hombrechtikon, Switzerland). The total chlorophyll content was 17.90 × OD_647_ + 8.08 × OD_665_.

### 4.11. Statistical Analysis 

All data are expressed as mean ± SD of three independent experiments. Statistical tests were performed using IBM SPSS (v21). Briefly, the Kolmogorov–Smirnov test and Levene’s test were used to check the normality of the data distribution and the homogeneity of variance of the data, respectively. Then, Student’s *t*-test and the Tukey–Kramer test were used for statistical analysis. The asterisk symbol (*) indicates significant difference at *p* < 0.05.

## Figures and Tables

**Table 1:** Effects of *Agrobacterium*-mediated gene transient expression.

Report Gene	Bacterial Strain	Number	The Positive Rate of Reporter Gene
*GFP*	GV3101	20	60.00%
*GUS*	GV3101	20	75.00%
*GFP*	AGL-1	20	65.00%
*GUS*	AGL-1	20	75.00%

**Table 2:** Effects of *Agrobacterium*-mediated TRV-based gene silencing systems in cassava.

Bacterial Strain	Number	The Positive Rate of Relative Transcript Level (<60%)	The Positive Rate of Albino Phenotype
AGL-1	8	75.00%	37.50%
GV3101	8	62.50%	12.50%

## Supplementary Materials

Supplementary materials can be found at https://www.mdpi.com/1422-0067/20/16/3976/s1.
