# Supplementary material for: Agrobacterium-Mediated Gene Transient Overexpression and Tobacco Rattle Virus (TRV)-Based Gene Silencing in Cassava
Source: Int J Mol Sci. 2019 Aug 15;20(16):3976. doi: 10.3390/ijms20163976 (PMC6719147; doi:10.3390/ijms20163976)
Supplement: Supplementary file 1 [file ijms-20-03976-s001.pdf]

Table S1. The primers used for vector construction.

Gene	Primer	Sequence	Vector construction
MePDS	MePDSF	CCGGAATTCGTGGAAAGGTGGC TGCGTGGA	MePDS-pTRV2
	MePDSR	CGCGGATCCTGCTTTCTCATCCA ATCTTG	

Table S2. The primers used for reverse-transcription PCR.

Gene	Primer	Sequence
MeEF1	QMeEF1F	TGAACCACCCTGGTCAGATTGGAA
	QMeEF1R	AACTTGGGCTCCTTCTCAAGCTCT
GFP	SQGFPF	ATGGTGAGCAAGGGCGAGGA
	SQGFPR	AGATCCGGTGGATCCAAGCT
GUS	SQGUSF	ATGTTACGTCCTGTAGAAAC
	SQGUSR	TCATTGTTTGCCTCCCTGCT

Table S3. The primers used for quantitative real-time PCR.

Gene	Primer	Sequence
MeEF1	QMeEF1F	TGAACCACCCTGGTCAGATTGGAA
	QMeEF1R	AACTTGGGCTCCTTCTCAAGCTCT
MePDS	QMePDSF	CAGCATCCTTCCGCAAT
	QMePDSR	TCCACGCAGCCACCTTT

Table S4. The primers used for VIGS detection.

Sample	Primer	Sequence
DNA	DpTRV1F	CTACCTGCAGATACGCCT
	DpTRV1R	ACTCACCCCCCAATAATC
DNA	DpTRV2F	GTTCATTTCATTTGGAGAGG
	DpTRV2R	AATGTCAATCTCGTAGG
cDNA	cDpTRV1F	GATAAGGAATTGAACCCG
	cDpTRV1R	CAAGGTGACTACGGCCA
cDNA	cDpTRV2F	TCCAGATAAGAAGGTGT
	cDpTRV2R	TTGTAACCATCATCACT

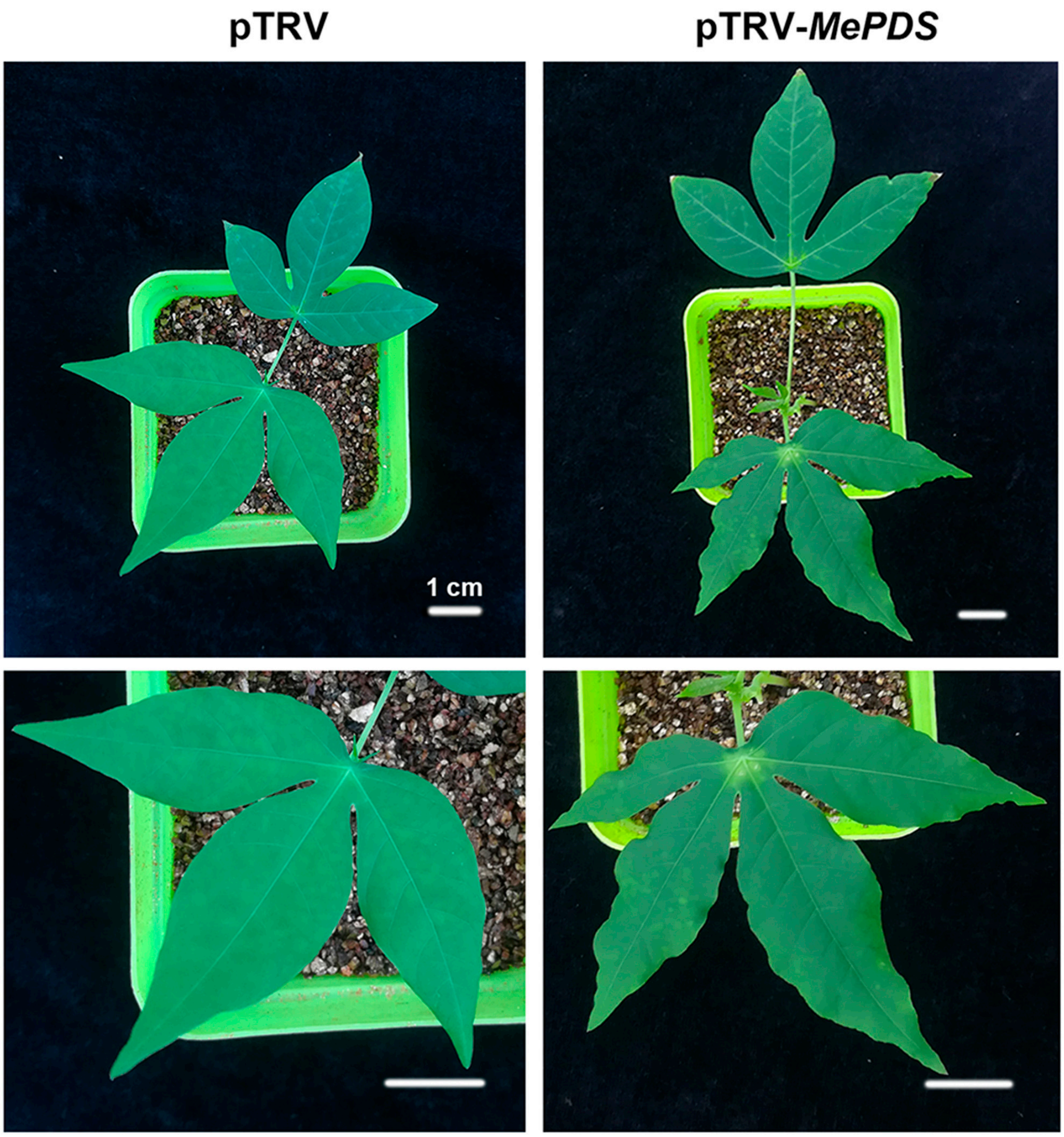

Figure S1. The albino cassava inoculated by *Agrobacterium* strain GV3101. The cassavas were inoculated with *Agrobacterium* strain GV3101 containing pTRV1 and pTRV2 vectors were seemed as mock, pTRV1 and pTRV2-*MePDS* were used to test the albino effect. 20 days after inoculation, the upper line shown the whole cassava plants, the underline shown the magnifying leaves. Bar = 1 cm.

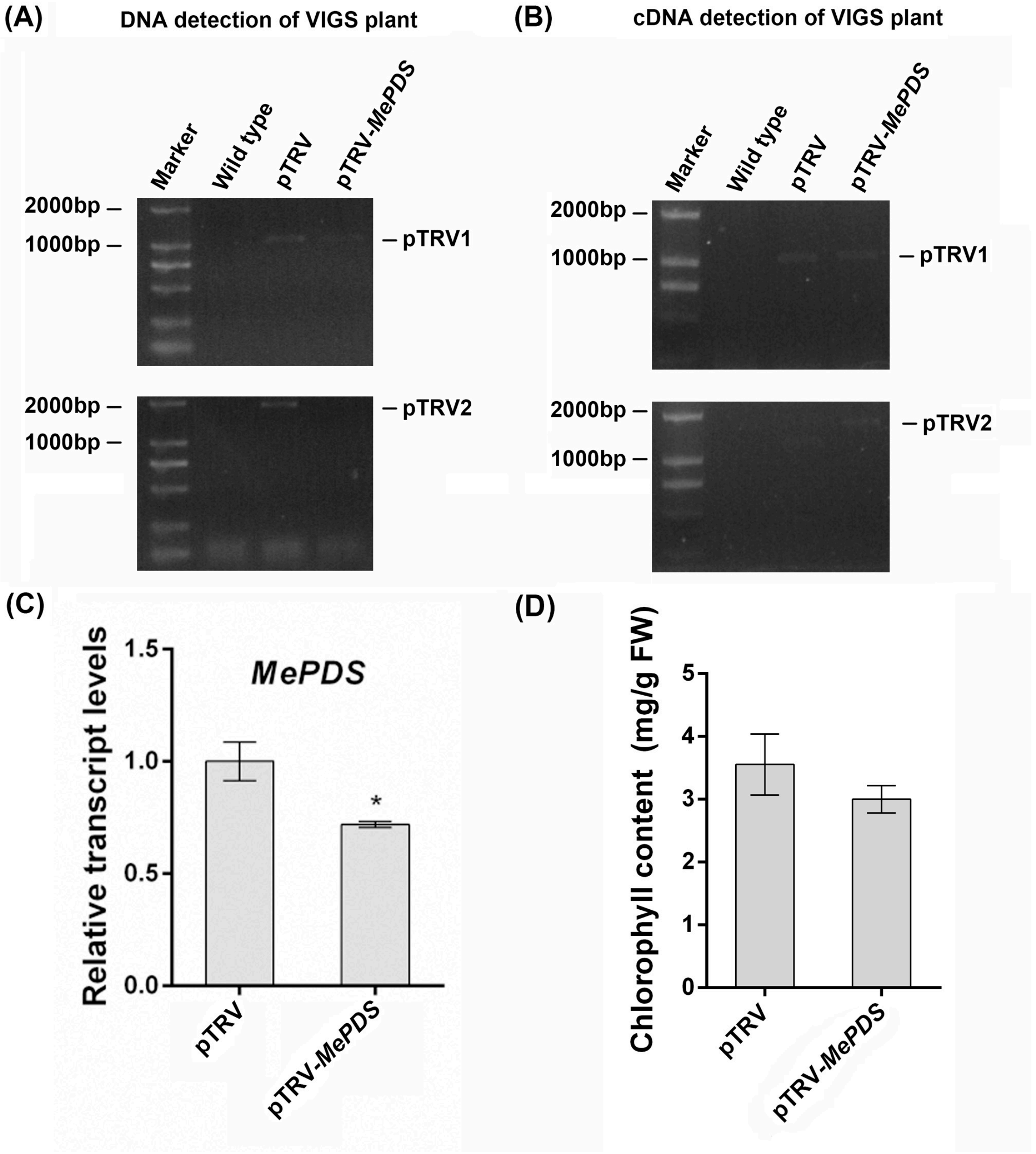

Figure S2. Detection of VIGS cassava plants inoculated by *Agrobacterium* strain GV3101. (A) PCR detection of pTRV1 and pTRV2 sequences from the genomic DNA of VIGS cassava plants. (B) RT-PCR detection of pTRV1 and pTRV2 sequences from the cDNA of VIGS cassava plants. (C) The relative transcript levels of *MePDS* of the infiltrated cassava plants. (D) The chlorophyll content in the infiltrated cassava plants. Asterisk symbols (*) of $p<0.05$ were shown by the calculation of student's t-test.
